# Circulating levels of non-muscle-specific miRNAs in response to acute muscle damage in rat

**DOI:** 10.1016/j.dib.2018.02.076

**Published:** 2018-03-07

**Authors:** Julien Siracusa, Nathalie Koulmann, Marie-Emmanuelle Goriot, Stéphanie Bourdon, Antoine Sourdrille, Sébastien Banzet

**Affiliations:** a Institut de Recherche Biomédicale des Armées, Brétigny sur Orge, France; b Ecole du Val de Grâce, Paris, France; c Institut de Recherche Biomédicale des Armées, Clamart, France; d INSERM UMRS-MD 1197, Clamart, France

**Keywords:** Circulating miRNA, Biomarkers, Muscle damage, Muscle toxicity

## Abstract

MicroRNA (miRNA) are found in numerous biofluids including blood and are considered a new class of biomarkers. In several animal models as well as in human diseases, they are interesting circulating markers of acute or chronic tissue injury. This article provides additional data related to a previous research article entitled “Circulating miRNAs as biomarkers of acute muscle damage in rats” by Siracusa et al. (2016) [1].

The data were obtained by RT-qPCR performed on plasma of rats exposed to acute muscle damage. The present set of data displays 45 non muscle-specific miRNA responses to acute, experimental muscle injury in healthy rats. They complement previous findings showing that circulating levels of miRNAs can be affected by muscle damage.

**Specifications Table**

**Table:** 

Subject area	*Biology*
More specific subject area	*Tissue damage, Biomarkers, Toxicology*
Type of data	*Figures*
How data was acquired	*RT-qPCR*
Data format	*Figures, data normalized with endogenous reference miRNAs and quantified*
Experimental factors	*RNA isolated from plasma after an experimental acute skeletal muscle injury*
Experimental features	*Muscle injury induced by notexin injection in soleus muscle of rats. Blood collected 6 h, 12 h, 24 h or 48 h later for miRNA analysis*
Data source location	*Clamart, France*
Data accessibility	*Data related to previously published article (Siracusa et al., 2016)*[1]

**Value of the data**

• These data describe plasma levels of non-muscle specific miRNA after an acute and massive experimental muscle injury.
• These data give insight into circulating miRNA response after skeletal muscle injury.
• These data are useful to researchers interested in miRNAs as biomarkers of tissue injury as well as scientists interested in circulating miRNAs in toxicology.

## 1 Data

**Fig. 1:**
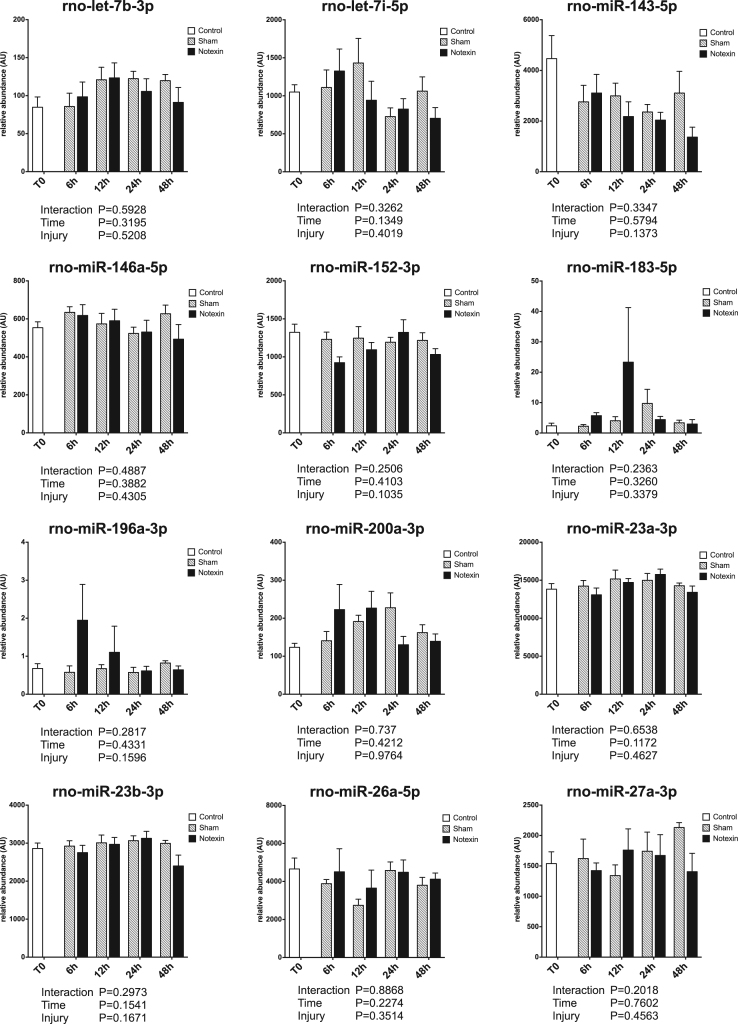

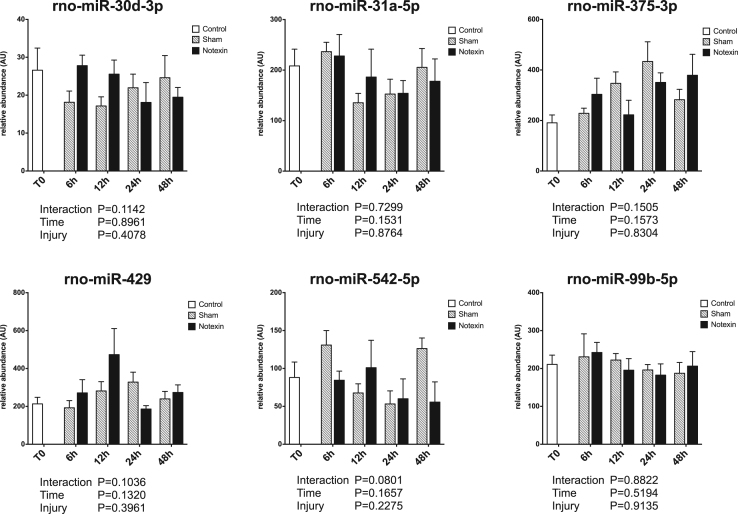
Plasma profiles of miRNAs in response to muscle acute muscle damage, with no significant effect of time or injury. Data are shown as mean and SE, and were analyzed with a two-way ANOVA.

**Fig. 2:**
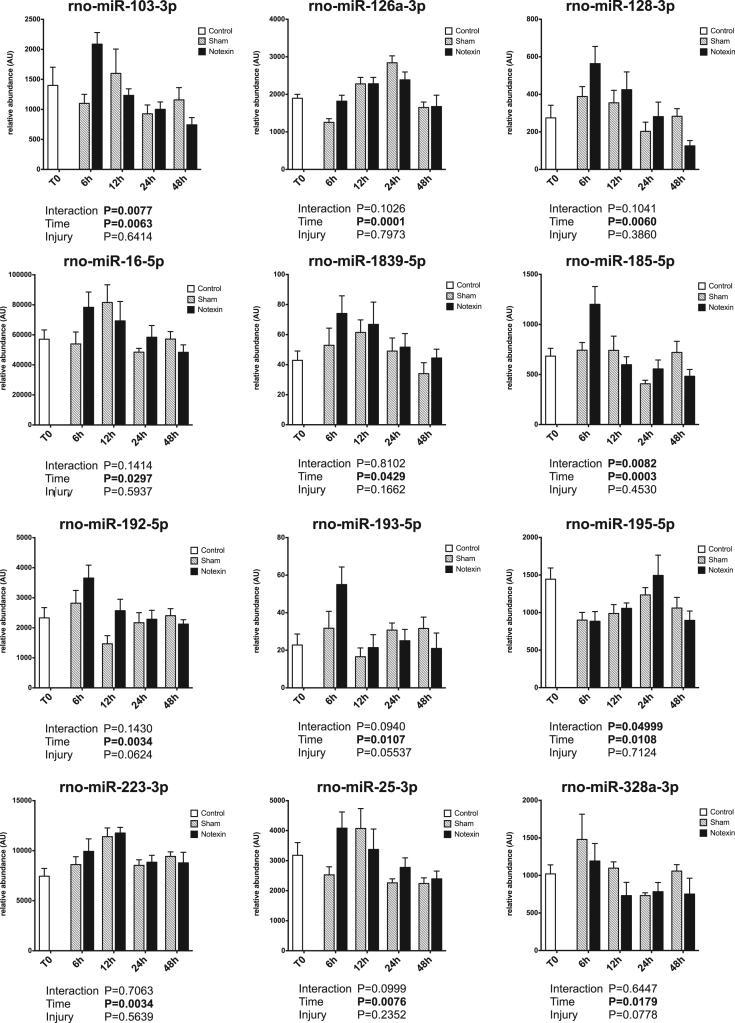

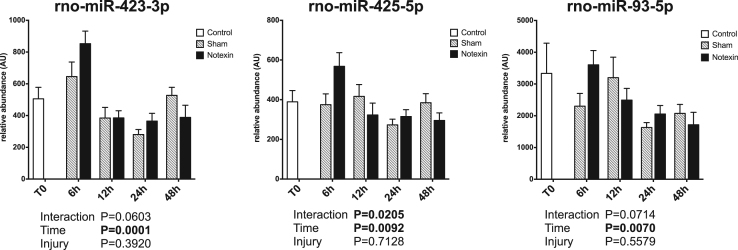
Plasma profiles of miRNAs in response to muscle acute muscle damage, with a significant effect of time but no significant effect of injury. Data are shown as mean and SE, and were analyzed with a two-way ANOVA.

**Fig. 3:**
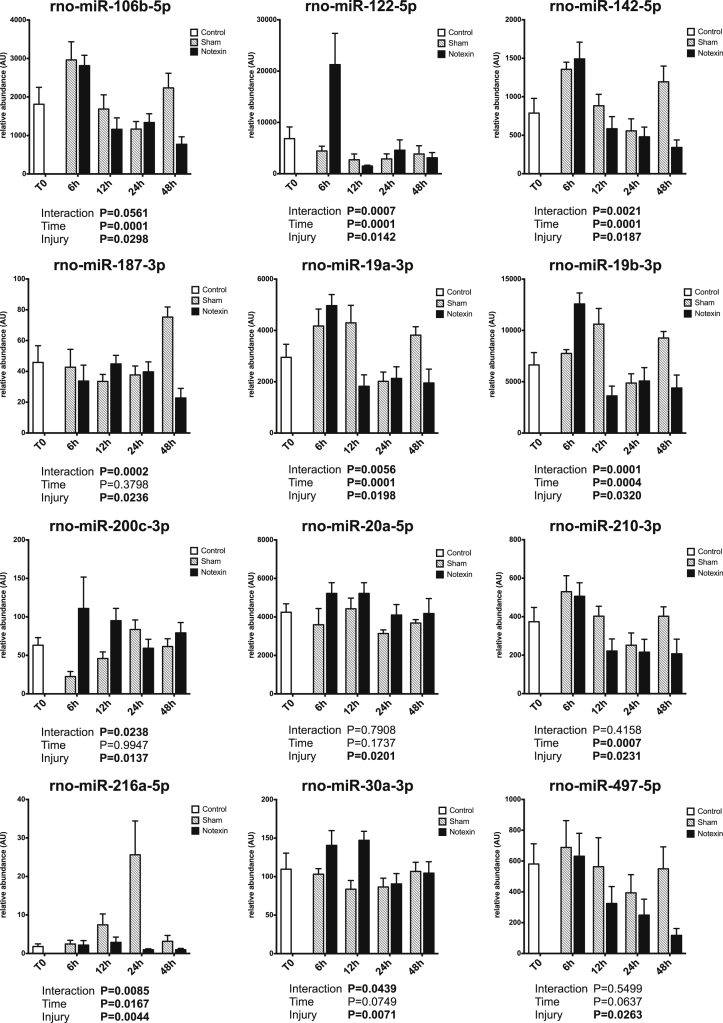
Plasma profiles of miRNAs in response to muscle acute muscle damage, with a significant effect of injury, with or without effect of time. Data are shown as mean and SE, and were analyzed with a two-way ANOVA.

Circulating miRNAs have been proposed to be useful biomarkers of tissue injury in various animal models as well as in human [2], [3], [4]. Skeletal muscle injury is a very common feature, ranging from mild exercise-induced muscle damage to severe rhabdomyolysis or muscle dystrophy. Upon injury, muscle specific miRNAs are released and their circulating levels increase significantly (up to 100 fold). Therefore, they are reliable markers of muscle damage [5]. Non muscle-specific miRNAs levels in plasma may also be affected by muscle damage. The present data set displays the early response of 45 miRNAs in rat plasma in the first 48 h after a severe muscle injury induced by injection of a myotoxic molecule (notexin) in soleus muscle (hindlimb), under surgery. Data are compared to sham operated rats. A profiling of over 700 miRNAs was first performed on pooled samples of each group. Then, a set of miRNAs was selected based on detectability and alteration in response to injury, and was measured on individual samples. Muscle-specific miRNAs results have been described elsewhere [1]. Fig. 1 displays miRNA profiles that were not significantly affected by the protocol. Fig. 2 displays miRNA profiles with a significant effect of time but no effect of the injury. Fig. 3 displays miRNA profiles with a significant effect of injury, with or without effect of time.

## 2 Experimental design, materials and methods

### 2.1 Animals

Two-month-old male Wistar rats were purchased from JANVIER Labs (Le-Genest-Saint-Isle, France). The experimentations were performed in accordance with the Helsinki Accords for Human Treatment of Animals during Experimentation and EU Directive 2010/63/EU for animal experiments. They received prior approval from local animal ethics committee (Comité d’Ethique pour l’Expérimentation Animale du Service de Santé des Armées).

The animals were randomly assigned to Notexin, Sham operated or Control group (*n* = 8). Rats were anesthetized with an intraperitoneal injection of ketamine (60 mg kg^−1^, Laboratoire Renaudin, Itxassou, France) and medetomidine (0.5 mg kg^−1^, Elanco, Greenfield, USA). Soleus muscle was surgically exposed, and 100 µL of notexin 10 µg mL^−1^ (Latoxan, Valence, France) were injected. In the sham groups, the same surgical gesture was applied with skin and fascia incision but without any injection in muscle tissue. A subcutaneous injection of buprenorphine (0.05 mg kg^−1^, Axience, Pantin, France) was performed after surgery to relieve pain. Then, an intraperitoneal injection of pentobarbital sodium (150 mg kg^−^^1^, Ceva Santé Animale, Libourne, France) was performed 6 h, 12 h, 24 h or 48 h later, blood was drawn from the abdominal aorta and rats were sacrificed by exsanguination. The control group was composed of healthy rats that underwent no surgery or anesthesia and were just sacrificed as described. Whole blood was collected in a K2E EDTA tube (BD Vacutainer, Plymouth, UK), centrifuged twice (2000 g, 10 min, 4 °C) and plasma was stored at − 80 °C.

### 2.2 RNA isolation

Total RNA isolation was achieved from 100 µL of plasma using mirVana Paris kit (Ambion, Austin, USA). A precipitation step was added: column elution was made with 180 µL sterile water, then 18 µL sodium acetate 3 M (Sigma-Aldrich, Saint-Louis, USA), 396 µL 100% ethanol, and 1 µL GlycoBlue (Ambion) were added. Precipitation was performed at − 20 °C during 20 min. The pellet was recovered after centrifugation (12,000 g, 15 min, 4 °C), washed with 70% ethanol, dried in a vacuum, and resuspended in sterile water (12 µL). Each sample was checked for contamination using a microvolume spectrophotometer.

### 2.3 Complementary DNA synthesis

cDNA were synthesized with the Universal cDNA Synthesis Kit (Exiqon, Vedbaek, Denmark) according to the manufacturer's recommendation. Because of the low RNA concentrations obtained (1–6 ng mL^−1^), a constant RNA amount couldn’t be easily used and a constant 1 μL volume was used as a template.

### 2.4 RT-qPCR

Pick-&-Mix microRNA PCR panels (Exiqon) were used [1], 500 µL 2× PCR Master mix were combined with 6.25 µL cDNA and 493.75 µL sterile water. Then, 10 µL were distributed in each well. qPCR were performed on a Light-Cycler 480 instrument (Roche, Manheim, Germany), with the cycling conditions recommended by Exiqon, including melting curve. Reference miRNAs (rno-miR-27b-3p, rno-miR-21-5p, rno-miR-151-3p, rno-miR-191a-5p, mmu-miR-351-5p, rno-miR-125a-5p, rno-miR-181a-5p) were selected using geNorm [6], and reached stability criteria. Quantification was performed as the geometric mean of the quantifications obtained with each reference miRNA.

### 2.5 Statistics

Results were log10-transformed and analyzed using a two-way ANOVA (injury/time) with Prism software version 6.01 (GraphPad, San Diego, CA). Results with a *p* value ≤ 0.05 were considered significant.
